# Automatic Detection and Quantification of Tree-in-Bud (TIB) Opacities from CT Scans

**DOI:** 10.1109/TBME.2012.2190984

**Published:** 2012-03-14

**Authors:** Ulas Bagci, Jianhua Yao, Albert Wu, Jesus Caban, Tara N. Palmore, Anthony F. Suffredini, Omer Aras, Daniel J. Mollura

**Affiliations:** 1 Center for Infectious Disease ImagingDepartment of Radiology and Imaging SciencesNational Institutes of Health Bethesda MD 20892 USA; 2 Department of Radiology and Imaging SciencesNational Institutes of Health Bethesda MD 20892 USA; 3 Naval Medical Center BethesdaMD 20889 USA; 4 Laboratory of Clinical Infectious DiseasesNational Institutes of Health Bethesda MD 20892 USA; 5 Department of Critical Care MedicineNational Institutes of Health Bethesda MD 20892 USA; 6 National Cancer InstitutesNIH Bethesda MD 20892 USA

**Keywords:** Computer-assisted detection (CAD), infectious diseases, lung, tree-in-bud (TIB), Willmore energy

## Abstract

This study presents a novel computer-assisted detection (CAD) system for automatically detecting and precisely quantifying abnormal nodular branching opacities in chest computed tomography (CT), termed *tree-in-bud* (TIB) opacities by radiology literature. The developed CAD system in this study is based on 1) fast localization of candidate imaging patterns using local scale information of the images, and 2) Möbius invariant feature extraction method based on learned local shape and texture properties of TIB patterns. For fast localization of candidate imaging patterns, we use ball-scale filtering and, based on the observation of the pattern of interest, a suitable scale selection is used to retain only small size patterns. Once candidate abnormality patterns are identified, we extract proposed shape features from regions where at least one candidate pattern occupies. The comparative evaluation of the proposed method with commonly used CAD methods is presented with a dataset of 60 chest CTs (laboratory confirmed 39 viral bronchiolitis human parainfluenza CTs and 21 normal chest CTs). The quantitative results are presented as the area under the receiver operator characteristics curves and a computer score (volume affected by TIB) provided as an output of the CAD system. In addition, a visual grading scheme is applied to the patient data by three well-trained radiologists. Interobserver and observer–computer agreements are obtained by the relevant statistical methods over different lung zones. Experimental results demonstrate that the proposed CAD system can achieve high detection rates with an overall accuracy of 90.96%. Moreover, correlations of observer–observer $(R^2=0.8848$, $p< 0.01)$ and observer–CAD agreements $(R^2=0.824$, $p< 0.01)$ validate the feasibility of the use of the proposed CAD system in detecting and quantifying TIB patterns.

## I. Introduction

Infectious lung diseases, such as novel swine-origin H1N1 influenza, tuberculosis (TB), etc., are among the leading causes of disability and death all over the world [1]–[3], [5]. Computed tomography (CT) examination of the lungs during acute respiratory tract infections has become an important part of patient care, both at diagnosis and monitoring progression or response to therapy. Although CT examination serves as a primary (imaging) diagnostic tool for assessing lung infections, visual analysis of CT images is restricted by low specificity for causal infectious organisms and a limited capacity to assess severity and predict patient outcomes [2].

Common CT findings associated with respiratory tract infections include tree-in-bud (TIB) nodularity, ground-glass opacities (GGO), random distribution of pulmonary nodules, linear interstitial/bronchovascular thickening, and consolidations [6]. Although none of these visual patterns are specific for one pathogen, the amount of lung volume exhibiting these features could provide insights into the extent or severity of infection. Among these patterns, TIB opacities, represented by thickened bronchial structures surrounded locally by clusters of 2–3 mm micronodules, are associated with inflammation of the small airways (bronchioles), such as in viral or bacterial bronchiolitis, and the increasing sizes of abnormal regions on CT can suggest the progression of disease [6]. Often considered to have a limited differential diagnosis-*M* TB infection, infection with nontuberculous mycobacteria, viral infection, cystic fibrosis, this pattern is recognized as a CT appearance of many different entities. Unlike the other imaging patterns such as GGO and consolidations, it is an extremely challenging task to detect and quantify the regions with TIB opacities due to interobserver variations and inconsistent visual scoring methods [2]. Therefore, an accurate method for detecting TIB is a critical in computer-assisted detection (CAD) schemes from chest CT. Although the correct diagnosis for TIB pattern is very important, it is also one of the most difficult tasks for radiologists because the contrast of lesions is often low and the disease patterns are very complex. All these limitations suggest that CAD could make a valuable contribution to the management of respiratory tract infections by assisting in the early recognition of pulmonary parenchymal lesions and providing quantitative measures of disease severity.

### A. Respiratory Tract Infections and TIB Patterns

**Fig. 1.**
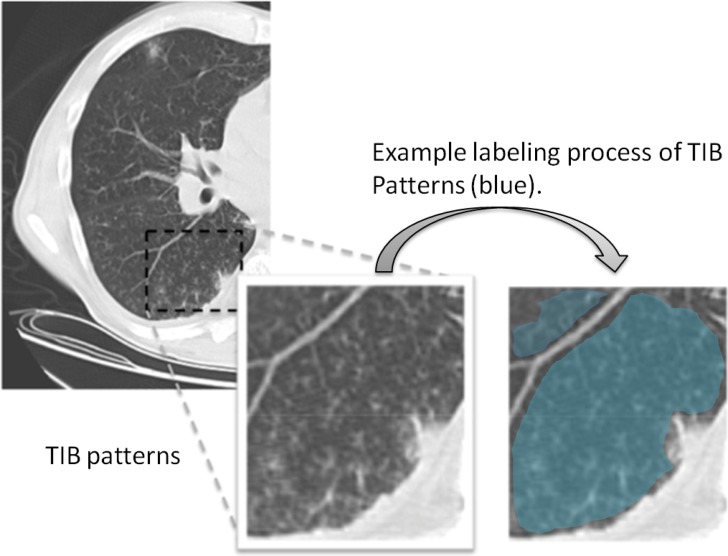
Single-axial CT slice with a significant amount of TIB patterns and an example labeling process of TIB patterns (blue) on the right lung are shown.

Respiratory tract infections, caused by viruses, bacteria, fungi, and parasites, are a major component of global infectious disease mortality. TIB patterns, in particular, usually represent the disease of the small airways such as infectious-inflammatory bronchiolitis as well as bronchiolar luminal impaction with mucus, pus, cells, or fluid causing normally invisible peripheral airways to become visible on CT [7]. Fig. 1 shows typical TIB patterns in a chest CT (Fig. 1 shows labeled TIB patterns with blue). As its name implies, this pattern resembles a budding tree in CT due to the branching opacities with adjacent centrilobular nodularity [2]. It is not specific for a single disease entity, but suggests pathology in the peripheral airways, which can be associated with air trapping or subsegmental consolidation in the surrounding alveolar airspaces. Because any organism that infects the small airways can cause a TIB pattern, pulmonary infections are its most common cause.

TIB is difficult to be detected with conventional CAD systems due to high complexity of their irregular shapes, as well as strong textural similarity of micronodules and thickened airways to other normal and abnormal lung structures. Currently, no reported CAD system is capable of automatically detecting a TIB pattern, therefore, which warrants a need for the development of such a system to improve the diagnostic decision process and quantitative measurement of respiratory tract infections. In this paper, we develop a new CAD system to evaluate respiratory tract infections by automatically detecting and quantifying TIB patterns on CT images.

### B. Our Contributions

The main contributions of this study are twofold. 1) A candidate selection method that locates possible abnormal patterns in the images. This process comes from a learning perspective such that the *size*, *shape*, and *textural characteristics* of TIB patterns are learned *a priori*. The candidate selection process removes large homogeneous regions from consideration which results in a rapid localization of candidate TIB patterns. The local regions enclosing candidate TIB patterns are then used to extract shape and texture features for automatic detection; 2) another novel aspect in this study is to extract Möbius invariant local shape features (i.e., Willmore energy-based features). Extracted local shape features are combined with statistical texture features to classify lung tissues. In addition, we also investigate the extraction and use of different local shape features as compared to the proposed shape features to facilitate local structure analysis. To the best of our knowledge, this is the first study that uses automatic detection of TIB patterns for a CAD system in infectious lung diseases. Since there is no published work on automatic detection of TIB patterns in the literature, we compare our proposed CAD system on the basis of different feature sets previously shown to be successful in detecting lung diseases in general. Early version of this study appeared in [3], and can be accessed in [8].

This paper is organized as follows. Section II explains the methods of the proposed CAD system. We discuss our proposed and conventional feature extraction methods in Section III. Next, we present the feasibility of the proposed CAD system by evaluating the detection and quantification performances in Section IV followed by a discussion and conclusion in Section V and Section VI, respectively.

## II. CAD Methodology

**Fig. 2.**
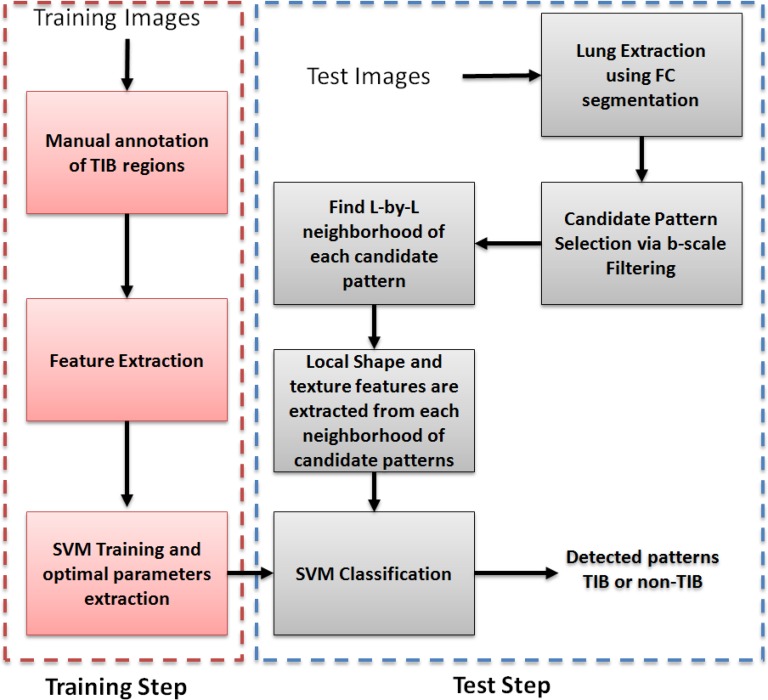
Flowchart of the proposed CAD system for automatic TIB detection.

The proposed CAD methodology is illustrated in Fig. 2. First, lungs are segmented from chest CTs. Second, we use locally adaptive scale-based filtering method to detect candidate TIB patterns. Third, segmented lung is divided into local patches in which we extract Möbius invariant shape features and statistical texture features followed by support vector machine (SVM) classification. We extract features from local patches of the segmented lung only if there are candidate TIB patterns in the patches. The details of the proposed methods are described in the following.

### A. Lung Segmentation

**Fig. 3.**
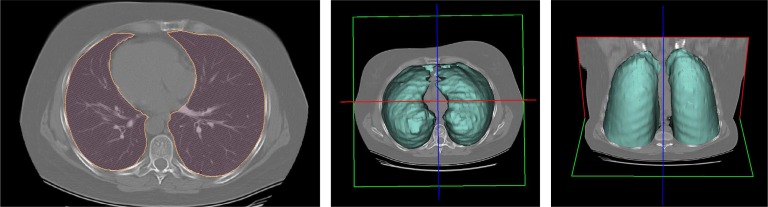
(Left) Axial single chest CT and its (middle and right) FC delineation correspondence are seen.

Prior to detection, segmentation is often the first step in CAD systems. In this study, fuzzy connectedness (FC) image segmentation algorithm is used to achieve successful delineations [9]. In FC framework, as illustrated in Fig. 3, left and right lungs are “recognized” by user-defined or automatically assigned seeds, which initiate FC segmentation. In this study, one seed per lung volume (i.e., left or right) is automatically set by only considering the locations of small intensity valued voxels inside the body region (see [9] for a detailed description of the use of FC in anatomy segmentation). Fig. 3 (middle and right) shows the resulting segmentation of the chest CT given on the left. Although we use FC algorithm to segment lung regions, there are many well-established lung segmentation methods in the literature [9]–[11], [29], and [41] such that they could possibly be used as well to accomplish the delineation step. In that sense, we do not have any strict restriction on the choice of segmentation algorithm prior to detection system as long as it successfully segments the lung regions.

### B. Learning Characteristics of TIB Patterns

The size/volume of a region occupied by a typical TIB pattern does not usually exceed a few ${\rm mm}^2/{\rm mm}^3$. Together with the fact that TIB pattern has a complex shape with varying intensities over discontinues branches (i.e., buds), TIB patterns have intensity characteristics with high variation toward nearby voxels (see Fig. 1). In other words, TIB patterns do not constitute sufficiently large homogeneous regions. Thus, TIB patterns are localized *only in the vicinity of small homogeneous regions*, and their boundaries have high curvatures due to the nature of its complex shape. In the next section, we use these two observations to extract novel characteristic features to detect TIB patterns.

**Fig. 4.**
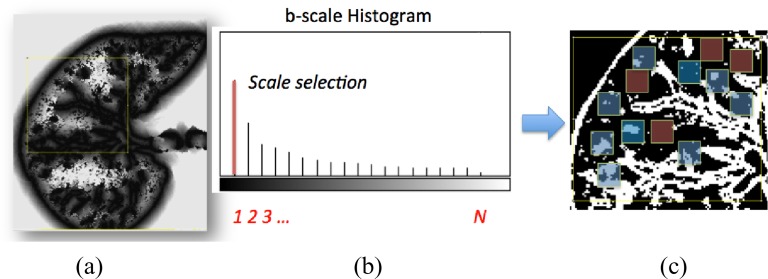
(a) b-scale scene. (b) Thresholding via selecting small-scale values only [i.e., a scale of 1 is selected as a threshold cutoff (red bar)]. (c) Thresholded b-scale scene; local regions without any b-scale pattern and with b-scale patterns are shown in red and blue, respectively.

Our candidate selection method comes from a learning perspective such that we assign every internal voxel of the lung a membership value reflecting the size (i.e., scale) of the homogeneous region that the voxel belongs to. To do this, we use a locally adaptive scale-based filtering method called ball-scale (or b-scale for short) [9], [16], [17]. The b-scale is the *simplest* form of a locally adaptive scale where the scene is partitioned into several scale levels. Every voxel in each scale is assigned the size of the local structure it belongs. For instance, voxels within the large homogeneous objects have highest scale values, and the voxels nearby the boundary of objects have small-scale values. Voxels on the boundary of objects have smallest scale values. Because of these observations, we conclude that TIB patterns constitute only *small b-scale values*; thus, it is reasonable to consider voxels with small b-scale values as candidate TIB patterns. Similarly, it is practical to discard voxels with high b-scale values from the candidate selection procedure. Figs. 2 (candidate selection) and 4(a) show selected b-scale regions as candidate TIB patterns. Once b-scale image correspondence of segmented chest CT is obtained as noted in Fig. 4(a), we only select small b-scale values via thresholding the large b-scale values [see Fig. 4(b)]. Justification of this selection procedure bases on the observations defined previously. Resultant candidate TIB patterns are shown in Fig. 4(c). We describe the computation of b-scale patterns and the details of the candidate selection process in the next section.

### C. Candidate Pattern Selection through B-Scale Encoding

There are several advantages to the local scale-based approach. For instance, boundary- and region-based representations of objects are explicitly contained in the scale-based methods. Based on continuity of homogeneous regions, geometric properties of objects (i.e., size information) can be identified, and this new representation is called scale images, i.e., b-scale, tensor-scale (t-scale), generalized-scale (g-scale) images [16], [18], [19]. The b-scale model has been shown to be extremely useful in object recognition [17], image segmentation [9], [41], filtering [16], inhomogeneity correction [20], and image registration [20], [21]. In this study, on the other hand, we show how to use b-scale encoding together with a proper scale selection method for detecting candidate abnormality patterns. The main idea in b-scale encoding is to determine the size of local structures at every voxel as the radius of the largest ball centered at the voxel within which intensities are homogeneous under a prespecified region-homogeneity criterion.

Although the conventional b-scale encoding method is well established for $nD$ images $(n\ge 2)$, we use 2-D b-scale encoding method in this study because low-resolution CT data do not allow continuous analysis of TIB patterns through low-resolution imaging direction. In the 2-D digital space $(Z^2,\nu)$, a scene ${\mathscr C}=(C,f)$ is represented by a pair where $C$ is a rectangular array of voxels, $\nu =(\nu_1, \nu_2)$ indicates the size of the voxels, and $f$ is a function that assigns to every voxel an image intensity value. A *ball* $B_{k}(c)$ of radius $k \ge 0$ and with center at a voxel $c \in C$ in ${\mathscr C}$ is defined by

$$B_{k}(c)=\left\{e \in C \Biggr \vert \sqrt{\sum_{i=1}^n (c_i - e_i)^2} \le k \right\}. \eqno{\hbox{(1)}}$$

The fraction of object is denoted by $FO_{k}(c)$ and indicates the fraction of the ball boundary occupied by a region which is sufficiently homogeneous with $c$. $FO_{k,\nu}(c)$ is defined as

$$FO_{k}(c)={\sum_{e \in B_{k}(c)-B_{k-1}(c)}W_\psi (\vert f(c)-f(e)\vert)\over \vert B_{k}(c) - B_{k-1}(c)\vert} \eqno{\hbox{(2)}}$$

where $\vert B_{k}(c)-B_{k-1}(c)\vert$ is the number of voxels in $B_{k}(c)-B_{k-1}(c)$ and $W_{\psi}$ is a homogeneity function [9]. In all experiments, we use a zero-mean unnormalized Gaussian function for $W_{\psi}$. The size of the local structure is estimated using appearance information of the gray-level images, i.e., region-homogeneity criterion; b-scale scenes contain rough geometric information. A detailed description of $W_{\psi}$ and $FO_{k,\nu}$ is presented in [9].

The b-scale algorithm works as follows: the ball radius $k$ is iteratively increased starting from one, and the algorithm checks for $FO_{k,\nu}(c)$, the fraction of the object containing $c$ that is contained in the ball. When this fraction falls below a predefined threshold, it is considered that the ball contains an object region different from that to which $c$ belongs [16]. This process is repeated for every voxel within the scene. Voxels are assigned their b-scale values discreetly from 1 to $r_{\rm max}$.^1^We set $r_{\rm max}=20$ in all cases due to the fact that size of the largest ball for one particular voxel rarely exceeds 15 voxels in length; see [16] for further details on the selection of maximum radius of the ball. In principle, b-scale partitions the scene into several levels based on the size of local structures from 1 to $r_{\rm max}$. Computing b-scale values for every voxel leads b-scale scenes as shown in Fig. 4(a). Note also that locally adaptive scale in regions with fine details or in the vicinity of boundaries is small, while it is large in the interior of large homogeneous objects.

## III. CAD Feature Extraction

Developing a successful CAD system for infectious lung diseases requires acquisition of representative features characterizing shape and texture of TIB patterns efficiently. Since TIB is a complex shape pattern consisting of curvilinear structures with nodular structures nearby, we propose to use local shape features (derived from geometry of the local structures) combined with gray-level statistics derived from a given local patch (i.e., local window with a predefined size).

**Fig. 5.**
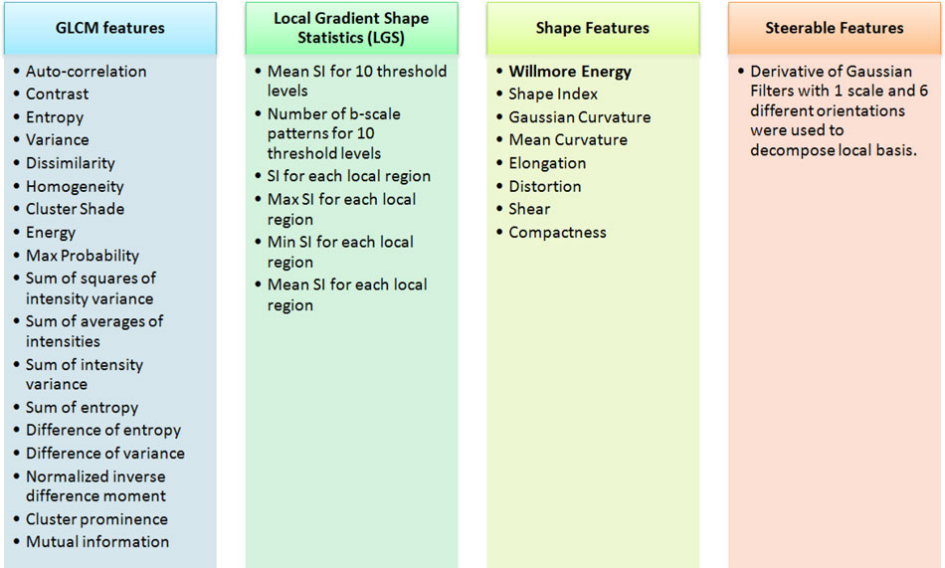
List of textural and shape features used to detect TIB patterns in the lungs.

The shape operator is the second-order invariant (or curvature) which determines the original surface. Since it is usually more convenient to work with scalar quantities rather than vectorial shape quantities, symmetric functions of local Hessian matrices are usually used to extract geometric meaning of the surface/shape of interest. Therefore, curvatures play an important role in the representation and recognition of intrinsic shapes. However, similarity of curvature values may not *necessarily* be equivalent to intrinsic shape similarities, which causes a degradation in recognition and matching performance. To overcome this difficulty, we propose to use Willmore energy functional [22] and several different affine invariant shape features parametrically related to the Willmore energy functional. While local shape features characterize the curvilinear and small nodular structures (via Willmore energy), gray-level features characterize background and foreground intensity variation with objects' pose and size for a given local window. Moreover, for comparison purpose, we use different feature sets previously shown to be successful in detecting lung diseases in general. Fig. 5 enlists all the features that we extracted for the proposed CAD system and for the experimental comparison. Details of extracted features are defined in the following.

### A. Willmore Energy and Shape Features

The Willmore energy of surfaces plays an important role in digital geometry, elastic membranes, and image processing [23]. It is closely related to Canham–Helfrich model [24], where a surface energy is defined as

$${\mathscr S}=\int_{\Sigma}\alpha +\beta (H)^2-\gamma KdA. \eqno{\hbox{(3)}}$$

where $\alpha, \beta$, and $\gamma$ are some constants, $H$ is the mean curvature vector on $\Sigma$ (area space), $K$ is the Gaussian curvature on $\partial \Sigma$ (boundary space), and $dA$ is the induced area metrics on $\Sigma$. This model is curvature driven, invariant under the group of Möbius transformations (in particular, under rigid motions and scaling of the surface) and shown to be very useful in energy minimization problems [25]. Invariance of the energy under rigid motions leads to conservation of linear and angular momenta, and invariance under scaling plays a role in setting the size of complex parts of the intrinsic shapes (i.e., corners, wrinkles, folds, etc.). In other words, the position, gray-level characteristics, size, and orientation of the pattern of interest have minimal effect on the extracted features as long as the suitable patch is reserved for the analysis. In order to have simpler and more intuitive representation of the given model, we simply set $\alpha =0$ and $\beta =\gamma =1$, and the equation turns into Willmore energy functional

$${\mathscr S}_w=\int_{\Sigma}(H^2-K)dA=\int_{\Sigma}\vert H\vert^2dA-\int_{\partial \Sigma}\vert K\vert ds \eqno{\hbox{(4)}}$$

where $ds$ is the length metric on $\partial \Sigma$. The resultant energy of a surface can be regarded as a function $H$ and $K$, and captures the deviation of a surface from local sphericity [22] such that a sphere has zero Willmore energy. Note also that the Willmore energy is always nonnegative. Since a homogeneity region that a typical TIB pattern appears is small in size, total curvature (or energy) of that region is high and can be used as a discriminative feature.

The main motivation in describing intrinsic shapes by Willmore energy is due to its ability to encode surface (i.e., image area in 2-D) with Möbius invariant features (translation, contrast, rotation, and inversion invariant). In addition to Willmore energy features that we adapt from Canham–Helfrich surface model, we have included seven different *local shape features*, which are parametrically related to Willmore energy formulation, into the proposed CAD system due to their some invariant properties and discriminative powers. Assume $\kappa_1$ and $\kappa_2$ indicate eigenvalues of the local Hessian matrix $H_e$ for any given local patch ${\mathscr L}$, the following shape features are extracted: 1) shape index (SI), 2) Gaussian curvature, 3) mean curvature, 4) elongation, 5) distortion, 6) shear, 7) compactness.

#### 1. Si

The *SI* is a statistical measurement and used to define intrinsic shape of the localized structure within the image [26], [27]. SI values are encoded as a continuous range of values between −1 and 1, with zero SI indicates saddle-like local structures, +1 and −1 SI values indicate umbilical minima and maxima (i.e., cap and cup, respectively), and midpoints of the two half-intervals (+0.5 and −0.5) indicate concave and convex parabolic or line-like structures (i.e., rut and ridge, respectively). SI can simply be computed through principal curvatures $(\kappa_1, \kappa_2)$ as follows:

$${\rm SI}={2\over \pi} {\rm arctan}\bigg({\kappa_1 + \kappa_2\over \kappa_1 - \kappa_2}\bigg) \in [-1,1] \eqno{\hbox{(5)}}$$

where $\kappa_1 \ge \kappa_2$. As suggested in [26], we obtain principal curvatures from the eigenvalues of the local Hessian matrix $(H_e)$ as

$$\left[\matrix{\kappa_1 \cr \kappa_2}\right] = {\rm eig}\left(H_e\right) = {\rm eig}\left(\left[\matrix{L_{xx} & L_{xy} \cr L_{yx} & L_{yy}} \right]\right) \eqno{\hbox{(6)}}$$

where $L_{xx}, L_{xy}=L_{yx},$ and $L_{yy}$ are second-order derivatives of local image patch ${\mathscr L}$, and ${\rm eig}()$ denotes eigenvalue decomposition. We choose to use ${\rm SI}$ because of its invariance property with respect to *rotation, absolute gray value,* and *translation*.

#### 2. Gaussian Curvature

Gaussian curvature ($K$) is an intrinsic measure and simply the product of the principal curvatures as $K=\kappa_1\kappa_2$ for a given point on a surface, equivalent to the determinant of local Hessian matrix $H_e$. Note that $K$ is unchanged even by bending the surface without stretching it, meaning that the Gaussian curvature is independent of the choice of unit normal and it gives three types of classified local shapes: elliptic shape ($K>0$), hyperbolic shape ($K< 0$), parabolic shape ($K=0$) with one of the $\kappa$ is equal to zero, planar shape ($K=0$) with both $\kappa$ are equal to zero. Gaussian curvature is *translation* and *rotation invariant*, but *not scale invariant*.

#### 3. Mean Curvature

Mean curvature ($H$) is an extrinsic measure and it describes the curvature as $H=(\kappa_1+\kappa_2)/2$. Unlike $K$, $H$ is defined in the distributional sense. Note that mean curvature measure is the trace of local Hessian matrix $H_e$. Mean curvature can be thought as a negative gradient (as a Laplacian) of the area functional due to its nice variational interpretation over the surface. This does not only give insights into the size of the local shape but also into the total symmetrical deviation from the sphere. Mean curvature is *translation* and *rotation invariant*, but *not scale invariant*.

#### 4. Elongation

Shape elongation is one of the basic shape descriptors and it indicates *flatness* measure of the local shape [28]. In this paper, we used the ratio of principal curvatures to measure elongation as $\kappa_2/\kappa_1$ with $\kappa_2\le \kappa_1$. Elongation measure is invariant with respect to a similarity transformation, and therefore, it is a robust feature that helps to identify curvilinear shapes. Elongation varies from $-1$ to $+1$, from hyperbolic to elliptic points.

#### 5. Distortion

Distortion is an algebraic quantity defined as the difference of eigenvalues (i.e., $\vert \kappa_1-\kappa_2\vert$) of the local Hessian matrix $H_e$. Distortion is a valuable image analysis property revealed by magnitude difference of principal curvatures. Distortion measure captures the deviation of principal curvatures, thus nonplanarity of a region. Together with Gaussian or mean curvature, distortion measure brings further information into encoding of local shape. Distortion measure is *translation* and *rotation invariant*, but *not scale invariant*.

#### 6. Shear

The shear is another algebraic distortion quantity defined as proportional to the normalized distortion: $(\kappa_1-\kappa_2)^2/4$. The physical information contained in the shear is basically the same as that of the distortion; it is related to distortion with powers of the difference of principal curvatures. Different than distortion, shear descriptor captures higher degree of nonplanarity of a region due to having more robustness against noise.

#### 7. Compactness

Compactness feature measures the similarity between shape of interest and a perfect ellipse, and is defined as $1/(4\pi \sqrt{\kappa_1\kappa_2})$. Note that this ratio is a dimensionless ratio between the area of the shape (1 for a normalized shape) and the area of the best ellipse fitting the shape. Note also that the compactness measure is invariant to affine transformations and parametrically related to Gaussian curvature.

**Fig. 6.**
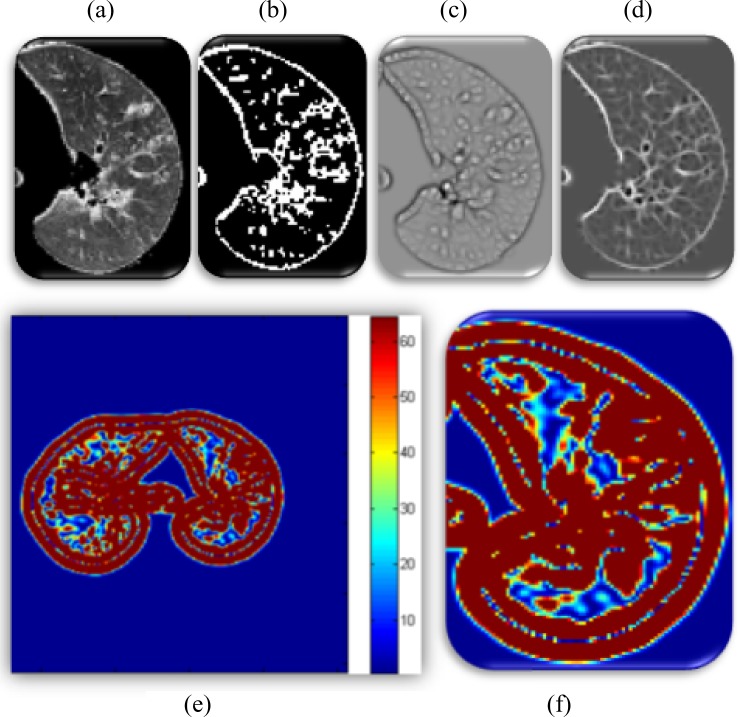
(a) Single-axial CT slice of the left lung. (b) Selected b-scale patterns. (c) Mean Curvature map ($H$). (d) Gaussian curvature ($K$). (e) Willmore energy map. (f) zoomed (e).

Given a single-axial CT slice of left lung, Fig. 6(b) indicates a thresholded (i.e., selected candidate patterns) b-scale scene encoded from the corresponding gray-level CT slice shown in Fig. 6(a). Furthermore, Fig. 6(c) and (d) shows mean and Gaussian curvature maps from which all the other local shape features are extracted, respectively. In addition, Fig. 6(e) and (f) shows Willmore energy maps using both mean and Gaussian curvature maps as formulated in (4) and shown in Fig. 6(c) and (d).

Based on the observation in training step where we analyzed the appearance and shape of TIB patterns, TIB patterns most likely occur in the regions inside the lung with high variability of intensity values over a small number of voxels and with certain size (i.e., a few millimeter in length). These observations (size and high intensity variation) facilitate one practically useful fact in the algorithm that, in the feature extraction process, we only extract features *if and only if* at least “one” *small* b-scale pattern exists in the local regions (i.e., blue local regions in Fig. 4).

### B. Local Gradient Shape Statistics (LGS) and Conventional Shape Features

We also explore the use of alternative local shape features as a comparison to Willmore energy-based features. Based on the observations of spatial properties of the selected candidate patterns, it becomes apparent that instead of using conventional high-dimensional feature extraction methods such as Gabor wavelets, steerable wavelets, etc., one may extract much fewer and more reliable statistical features to discriminate the pattern of interest. Motivated from the fact that TIB patterns consist of numerous small (or micro-) nodules nearby the main curvilinear structure and those small structures have varying opacities, the location and distribution of those small structures can be obtained by simple thresholding method which has been popular in estimation for more than two decades [30]. However, since the opacities are varying through different nodular structures, it is challenging to find an optimum threshold value. Therefore, instead of using one single threshold level, we empirically choose $n=10$ different threshold levels ($\lambda_j$) to obtain local statistics of those structures in a hierarchical way, where $\lambda_j=10j$, $1\le j \le 10$ [31]. This process is named LGS because we extract different statistical measurements in gradient of the images. Note also that we confine ourselves into the local patches where at least one b-scale pattern occupies.

**Fig. 7.**
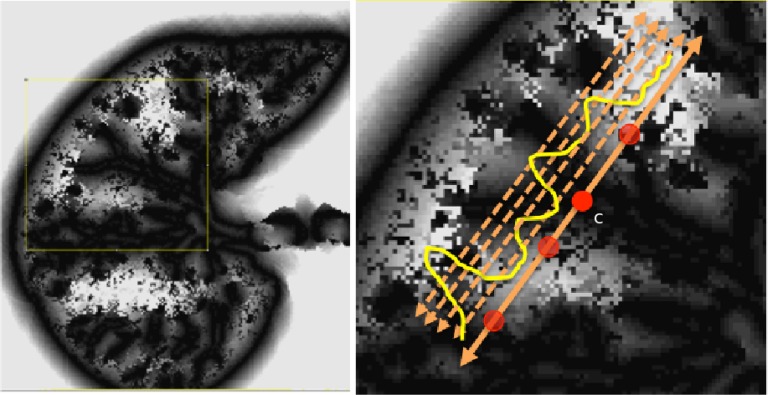
Local gradient maxima above different thresholds are shown.

To obtain shape statistics over local patches, we use gradient fields because boundary information can be used much more effectively in that sense. Fig. 7 shows an example thresholding process over a candidate TIB pattern centered at $c$ (only for four levels are shown for demonstration purpose). After different threshold levels are applied over the local regions of b-scale images, resultant thresholded local patches are used to extract the following features: mean SI values of the local patch for each thresholding level (one feature), and the number of b-scale patterns left after thresholding process (one feature). Since we use ten different thresholding levels, we extract 20 features totally. Moreover, for a local region centered at a voxel $c$ of a candidate TIB pattern, we extract one global feature as an SI value of the voxel $c$, three features as the maximum, minimum, and mean SI values over the local region prior to thresholding. Therefore, a total of 24 features (LGS+SI) are extracted from a typical local patch to be used in CAD system [4]. Although $n$ and $\lambda_j$ are chosen empirically based on the observations of shape and textural characteristics of normal and TIB patterns during the training step, one may propose to use cross validation, control of the global and local false discovery rate, and uncertainty principles to decide those parameters near-optimally [30].

### C. Conventional Texture Features

*Steerable Features:* It has been well documented in the literature that decomposition of images by using basis functions localized in spatial position, orientation, and scale (e.g., wavelets) are extremely useful in object recognition and detection [32], [33]. Since steerable filters are *rotation and translation invariant*, they accurately represent the underlying image structure [34]. In this study, we use steerable derivative of Gaussian filters to decompose local regions around each candidate pattern into several oriented basis. These basis are used as features in voxel-wise classification for TIB identification. We extract steerable features (i.e., directional derivatives) from one scale and six different orientations.

*Gray-Level Co-Occurrence Matrix (GLCM) Features:* Spatial statistics based on GLCM [35] are shown to be very useful in discriminating and quantifying patterns pertaining to lung diseases. As texture can give a lot of insights into the classification and characterization problem of poorly defined lesions, regions, and objects, we combine our proposed shape-based invariants with Haralick's popular GLCM-based features [35]. We extract 18 features from each local patch including autocorrelation, contrast, entropy, variance, dissimilarity, homogeneity, cluster shade, energy, maximum probability, sum of squares of variance, sum of averages, sum of variance, sum of entropy, difference of entropy, difference of variance, normalized inverse difference moment, cluster prominence, and mutual information. Readers are encouraged to refer to [35] for further details on these well-established features in machine learning, and [12]–[15] for particular CAD systems in identification of lung abnormalities from CT scans in general.

## IV. Experiments and Results

### A. Data

Laboratory confirmed (with pathology identification tests) 39 CTs of human parainfluenza (HPIV) infection and 21 normal lung CTs were collected for the experiments. All patients were imaged at our institution using a 64-detector row Philips Brilliance or a 320-detector row Toshiba Aquilion CT scanner. The noncontrasted chest CT studies were performed at end inspiration with 1.0 or 2.0 collimation obtained at 10- or 20-mm intervals from the base of the neck to upper abdomen with a tube voltage of 120 kV and a current of 200–320 mA depending on the subject's weight. Imaging data were constructed to 512 × 512 matrices with slice thickness of 5 mm. The in-plane resolution was affected by patients' size and varied from 0.62 to 0.82 mm. All 60 CT scans (both HPIV and normal) were collected from different subjects (no multiple scans from subjects).

### B. Training Step

A well-trained radiologist [with more than nine years experience (DMJ)] carefully examined the complete scan (i.e., 60 CTs) and labeled the lung regions as normal and abnormal (with TIB patterns) (see Fig. 1). As many regions as possible showing abnormal lung tissues from 39 HPIV patients were labeled (see Table I for details of the number of regions used in the experiments). Those 39 patients do not include only TIB opacities, but also GGO, nodules, consolidations, and linear thickening such that only TIB regions are labeled in training step. Note also that the control group consisting of 21 subjects with no observed lung abnormalities was constructed and lung tissues pertaining to this group were labeled carefully.

**Table I:** Accuracy ($A_z$) of the CAD System With Given Feature Sets

Features	Dimension	Patch Size	# of patches (TIB)	# of patches (Normal)	Area under ROC curve: $A_{z}$
Shape & GLCM	8+18=26	$17\times 17$	14144	12032	0.8991
Shape & GLCM	8+18=26	$13\times 13$	24184	20572	0.9038
Shape & GLCM	8+18=26	$9\times 9$	50456	42924	0.9096
Shape	8	$17\times 17$	14144	12032	0.7941
Shape	8	$13\times 13$	24184	20572	0.7742
Shape	8	$9\times 9$	50456	42924	0.7450
Steer. Wavelets& Shape	$6\times 17\times 17+8=1742$	$17\times 17$	14144	12032	0.7846
Steer. Wavelets& Shape	$6\times 13\times 13+8=1022$	$13\times 13$	24184	20572	0.7692
Steer. Wavelets& Shape	$6\times 9\times 9+8=494$	$9\times 9$	50456	42924	0.7908
SI & LGS	24	$17\times 17$	50456	42924	0.7620
SI & LGS	24	$13\times 13$	50456	42924	0.7510
SI & LGS	24	$9\times 9$	50456	42924	0.7230
Steer. Wavelets	$6\times 17\times 17=1734$	$17\times 17$	14144	12032	0.7571
Steer. Wavelets	$6\times 13\times 13=1014$	$13\times 13$	24184	20572	0.7298
Steer. Wavelets	$6\times 9\times 9=486$	$9\times 9$	50456	42924	0.7410
GLCM	18	$17\times 17$	14144	12032	0.7163
GLCM	18	$13\times 13$	24184	20572	0.7068
GLCM	18	$9\times 9$	50456	42924	0.6810

In the training step, we also explored how the number of b-scale patterns change for normal and diseased subjects. Our observations from detail analysis in candidate selection part showed that only 21–40% of the segmented lung volumes were chosen as candidate TIB patterns. This interval was subject to change based on the severity of the diseases. For patients without having infections (i.e., control group), for instance, the percentage of the candidate regions was smaller than the patients with infections; therefore, an increase in the amount of small-sized b-scale patterns is observed. In any case, local scale could be used as a quantitative measure validating the sensitivity and specificity of the classification rates as we describe it in Section IV-E.

### C. Visual Grading Scheme

**Fig. 8.**
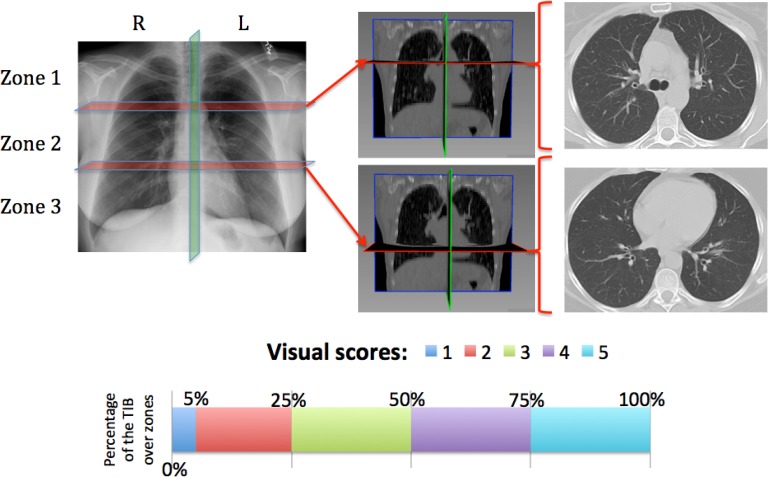
First row: lungs are divided into (left) three zones. Rough anatomical locations separating zones are shown in (middle) coronal and (right) axial CT slices, respectively. Second row: visual grading scheme.

Occurrences of TIB abnormality and normality of subjects were noted for each CT scan. To analyze existence and severity of abnormality as well as normality of subjects, a visual grading system was adapted from studies examining CT findings in other infections [36]–[38]. Each lung was divided into three zones (for a bilateral total of six) as shown in Fig. 8. Zone 1 included the apex to the carina. Zone 2 extended from the tracheal carina to the left atrium's junction with inferior pulmonary veins. Zone 3 included the remainder of the lungs below the level of the inferior pulmonary veins atrial junction. A severity score (0 to 5 such that 0 indicates no abnormality) was assigned to each zone based on the percentage of the zone occupied as listed in Fig. 8 (second row). A total score was also extracted by considering all zones during visual grading. Consensus visual scores^2^Consensus visual scores were obtained when AW and DJM scored the cases by mutual agreement. from participating radiologists [one with more than nine years of experience (DMJ) and one with more than one year of experience (AW)] on a scale of 0–5 over lungs were recorded and compared with computer scores (of the proposed CAD system). Following the same visual scoring scheme, another participating radiologist [with more than seven years of experience (OA)], who was blinded to the consensus visual scores previously obtained, was involved in the visual grading process to provide information on interobserver variability.

### D. Quantitative Evaluations

To measure and evaluate the detection capabilities of a CAD system quantitatively, the area under the receiver operator characteristic (ROC) curves is often used [39]. After the proposed CAD system was tested via twofold cross validations with labeled dataset, we presented ROC curves of the system performances.

Table I summarizes the performance of the proposed CAD system as compared to other feature sets. The performances are reported as the areas under the ROC curves $(A_z)$. Note that proposed shape features (i.e., Willmore energy and parametrically related local shape features) alone are superior to other methods even though the dimension of the proposed shape feature is only 8. The best performance is obtained when we combine the proposed shape and GLCM features. This is to be expected because spatial statistics are incorporated into the shape features such that texture and shape features are often complementary to each other. On the other hand, compared to the proposed shape features, the LGS and SI features have lower detection rates because they are not affine (and Möbius) invariant and eventually having difficulty in appreciating the large amount of details of TIB patterns. Another reason is that there is no optimal choice of thresholding process and this may yield less remarkable statistical measurements over local patches. However, the LGS and SI features alone perform better than the high-dimensional conventional features similar to the proposed shape features. This result itself suggests the use of local shape features and their adapted extensions in detection of TIB patterns.

**Fig. 9.**
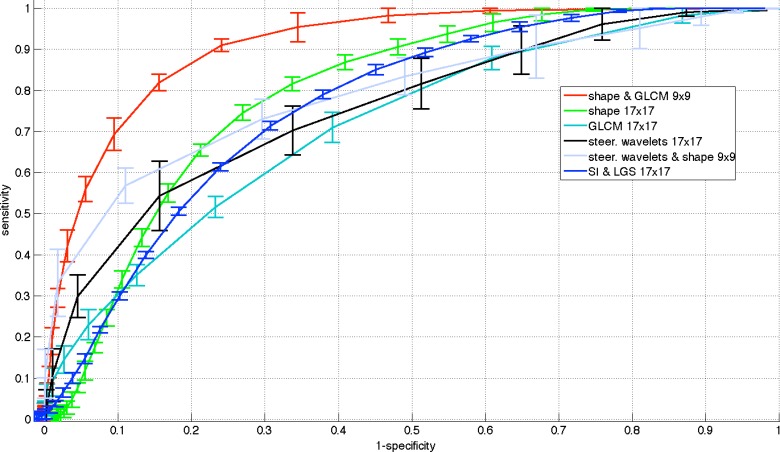
Comparison of CAD performances via ROC curves of different feature sets.

In what follows, we selected the best window size for each feature set and plotted their ROC curves all in Fig. 9. Superiority of the proposed shape features is clear in all cases. To have a valid comparison, we repeated candidate selection step for all the methods because we observed that the CAD performances of compared conventional feature sets had much lower accuracies if the candidate selection part was not applied (i.e., proposed method's accuracy was decreased to $A_z=0.6803$, while the best result of all compared methods were decreased to $A_z< 0.5281$). To show whether the proposed method was significantly different than the other methods, we compared the performances through paired *t*-tests. *p*-values of the tests indicate that none of the feature set are significantly correlated with the proposed CAD features such that highest and smallest *p*-values are reported as 0.0195 ($p< 0.05$) and 0.0053 ($p< 0.01$), respectively.

### E. Qualitative Evaluations

Visual scoring by radiologists still lies at the heart of diagnostic decisions, and often used as a validation tool. In this section, we explore the correlation between computer score (i.e., CAD score) and visual scores by participating radiologists. Furthermore, we investigate the effectiveness of the proposed method's ability to *roughly* discriminate normal and diseased patients by only considering the size of the structures pertaining to lung anatomy.

**Fig. 10.**
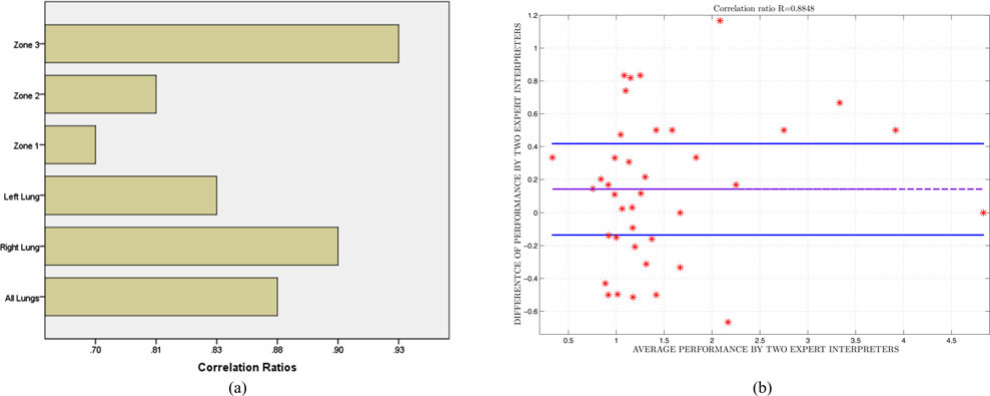
(a) Interobserver agreements given by Pearson product–moment correlation ratios. (b) Bland–Altman scatter plot is drawn for analysis of variability of change of scores between observers.

Based on the visual grading scheme explained in Section IV-C, we compared the consensus reading of two expert observers (AW and DJM) to another expert observer (OA), who was blinded to the consensus scores. We used Pearson product–moment correlation coefficients to determine interobserver agreement over each zone, left, right, and all lung volumes. The reported correlation ratios are shown in Fig. 10(a). Note that interobserver agreement correlation values for all TIB measurements were high for all zones and the lung. The lowest agreement seen on the zone 1 may be because subtle abnormalities in this zone may have been given greater visual assessment variance among the observers. Nevertheless, an overall correlation coefficient of $R^2=0.8848$ $(p< 0.01)$ indicates an excellent agreement on the existence of TIB patterns.

We further analyzed the variability of change of scores of expert radiologists for each subject. For this, we constructed Bland–Altman plot [40] where the limits of observer agreements were indicated by ${\rm bias}$ ± $1.96 \,{\rm std}$ (bias: average difference, std: standard deviation). In Bland–Altman plot, the difference of the performances was plotted against the average of the performances as shown in Fig. 10(b). It was noted from this figure that the largest disagreement of the scoring between observers never exceeded 1.2 over six levels of scores, validating the good agreement shown by Pearson product–moment correlation coefficients.

**Fig. 11.**
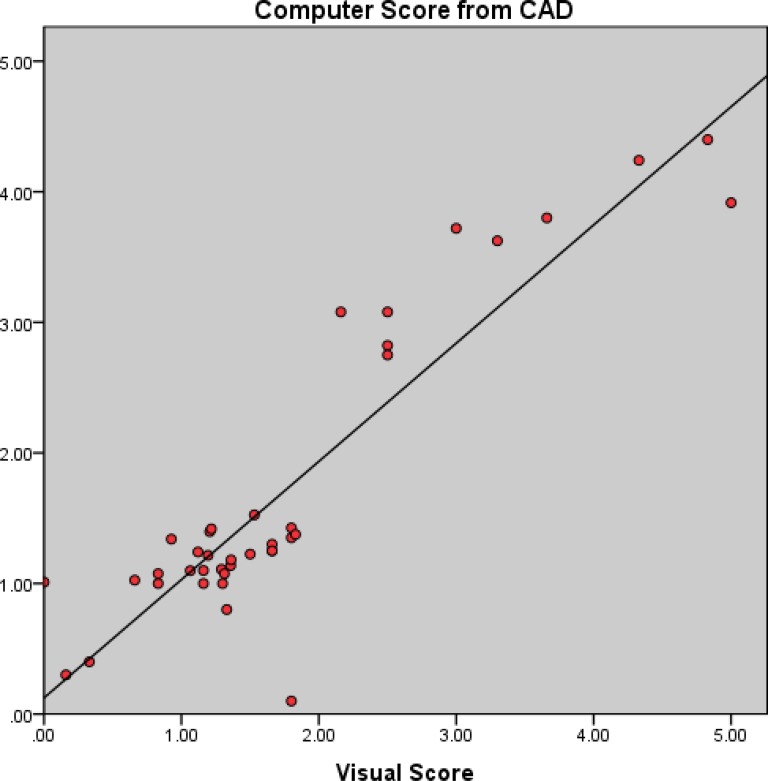
Visual grading versus computer evaluation. A Pearson product–moment correlation of $R^2=0.824$ is reported.

**Fig. 12.**
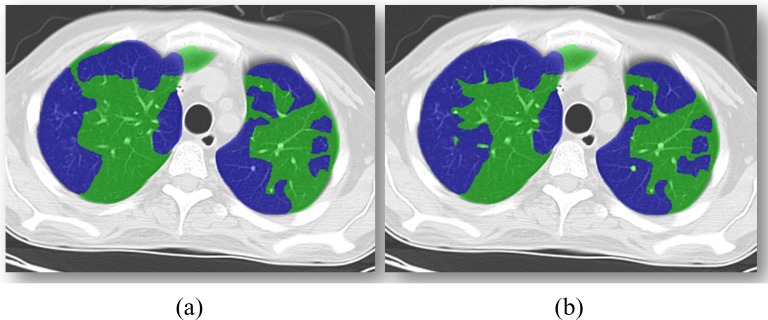
Random slice from an example HPIV case for quantification is shown. (a) Expert annotation of TIB (blue) and non-TIB (green) regions. (b) Computer quantification of TIB (blue) and non-TIB (green) regions.

To obtain an *overall computer score* from the proposed CAD system, on the other hand, TIB regions detected by the CAD system were first labeled automatically during the detection process. Then, a computer score was calculated by averaging the volume occupied by the labeled TIB regions over the whole lung volume. Calculated computer score was then normalized to fit the visual grading scheme explained in Section IV-C. Linear regression model was fitted to all subjects' scores both from computer and the consensus scores of the participating radiologists (DMJ and AW) and Pearson product–moment correlation coefficient was computed for this model. A scatter plot of the linear regression model and the computer–observer agreement correlation is shown in Fig. 11. It is clear from this plot that visual and quantitative assessments correlate well as indicated by the Pearson product–moment correlation of $R^2=0.824$ $(p< 0.01)$. Finally, we illustrate an example of TIB and non-TIB region classification by expert annotation and computer quantification by our proposed method in Fig. 12(a) and (b), respectively.

**Fig. 13.**
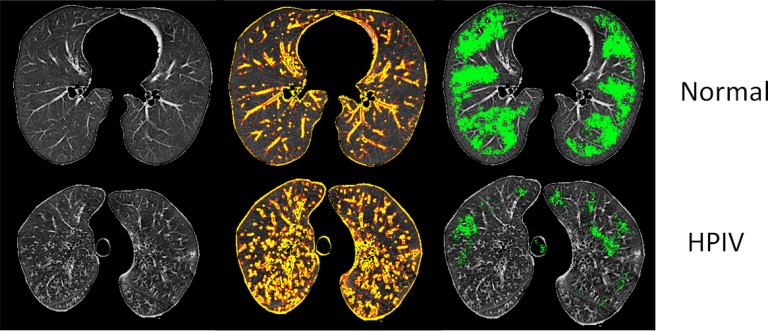
First column: segmented lungs. Second column: patterns occupying in small homogeneous regions. Third column: patterns occupying in large homogeneous regions. Note that patient with HPIV shows large number small-scale patterns, and less number of large-scale patterns.

*Scale-based analysis:* In addition to visual scoring scheme, we also show the effectiveness of the proposed scale-based method on quantification of the disease extent and identification. Scale-based analysis of the regions occupied by TIB patterns is illustrated in Fig. 13. A CT slice of a patient with HPIV shows fewer large homogeneous regions (green) with respect to a normal control. It also shows a greater number of small homogeneous regions (yellow and red).

**Fig. 14.**
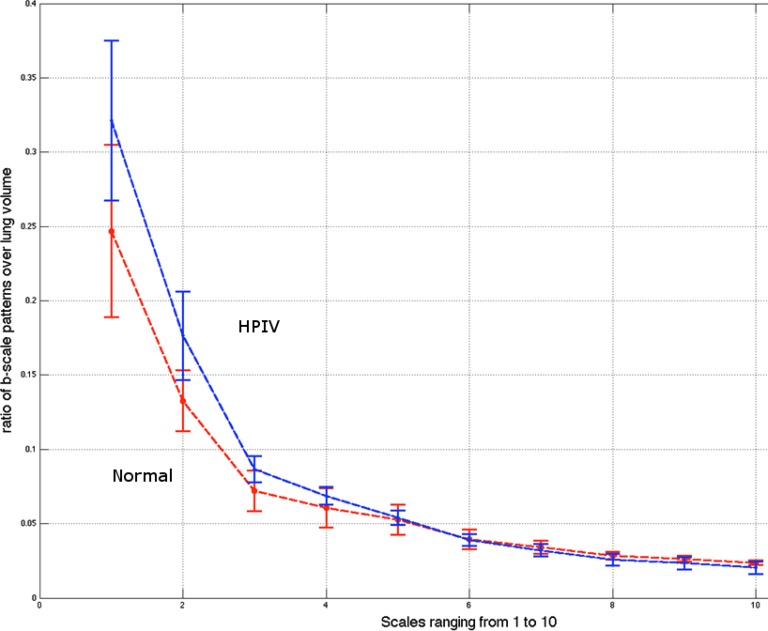
Diseased with HPIV (in blue). Normal controls (in red). Curves show mean and standard deviation values of number of patterns in scales 1 to 10. Patients with HPIV show more small-scale patterns and fewer number of large-scale patterns.

Fig. 14, on the other hand, shows deviations of the number of scale patterns over normal and disease cases. For each scale (from 1 to 10), we recorded the average number of b-scale patterns. As readily seen from both curves, the existence of TIB patterns was indicated through the small number of highly homogeneous regions (i.e., small number of large b-scale patterns) and large number of less homogeneous regions (i.e., large number of small b-scale patterns). This figure validated the qualitative results shown in Fig. 13. The difference between two curves was at statistically significant level $(p< 0.01)$.

### F. SVM Classification, Computational Cost, and Algorithm Details

All programs used in this study were developed using gcc 4.5 (Copyrigth (C) 2010 Free Software Foundation) on a Linux platform (Pardus), and all statistical computations were processed in R (Version 2.12.2) and MATLAB (Copyright (C) 2010 Mathworks). All the programs were executed on an Intel (R) Core(TM) i7 CPU 930 at 2.80 GHz with 12 GB RAM workstation. While segmentation of lung regions from CT scans takes only about 10 s, the b-scale encoding algorithm takes a couple of minutes (average 2 min, at most 5 min). The time required to compute b-scale scenes changes from patient to patient due to different number of slices in CT scans. Details of the computational cost analysis for segmentation of lungs, and feature extractions for particular algorithms are enlisted in Table II.

**Table II:** Computational Cost Analysis of the Methods

Method	Avg. Time
Segmentation of lung regions	10 sec
b-scale encoding	2 min
Proposed features	8-9 min
Shape features	3 min
GLCM	5-6 min
Steer. wavelets	40-45 min
Steer. wavelet+shape	43-48 min
SI + LGS	8-10 min

A further feature selection method such as a principal component analysis might be used to reduce the dimension of steerable features that we used only for comparison purposes. Note that the proposed features are having a small number of dimension per local patch; there is not necessarily an additional feature selection method needed; hence, it is outside the scope of this paper.

Briefly, the whole dataset was randomly divided into training and test sets of 30 CT scans (20 HPIV-10 Normal versus 19 HPIV-11 Normal). Parameters of the SVM classifier were learned based on the CT scans pertaining to training set. SVM regression was based on pixel-wise classification [42]. Followed by feature extraction step, the trained SVM classifier was applied to the test set. Note also that we have used twofold cross-validation technique for training and testing; therefore, we changed the role of training and testing dataset in the second fold. We also noticed that there was no significant changes in training and test performances of SVM classifications if twofold cross validation was changed into *n*-fold cross-validation system with $n>2$. In addition, we have used Efron's bootstrap [43] method (i.e., repeating the experiments 100 times based on the actual data) to assess the variability of the estimated classifications derived from SVM regressions, and provide confidence intervals for ROC curves.

We used radial basis functions as kernel of SVM, and set to epsilon parameter of SVM as 0.1 [42]. Resulting SVM values of pixels are ranging from 0 to 1. This value indicates the likelihood of a local patch belonging to a certain class (TIB or non-TIB); low ratings indicate a non-TIB region, and high ratings indicate a TIB region. Soon after the SVM values were computed for the entire lung, we changed the cutoff values of SVM (0.5 as default) several times to obtain ROC curves.

## V. Discussion

In this paper, we studied a very particular, yet important, pattern of lung abnormality observed in chest CTs. Our proposed detection system is tuned to detect TIB regions from non-TIB regions; therefore, a multiclass classifier (with specifically tuned detection filters for each abnormality class) might be needed as an extension of this study to detect as much abnormality as possible in a whole system. Although such a system will bring its unique challenges into the CAD platform, it would be a valuable second opinion tool for radiologists. As a further step, we are currently investigating combining different imaging patterns pertaining to lung abnormalities as well as clinical laboratory information into our CAD system.

One question arises as to the use of high-resolution CT (HRCT) scans instead of conventional CT scans in detecting TIB patterns, as well as the effect of using HRCT scans in this process. Although HRCT scans appreciate detection of small nodular patterns, they have more noise and lungs might not be fully covered due to large gaps between slices (i.e., 10–30 mm). Furthermore, at our institution and in many other institutions, the protocol for acute pulmonary infection is 5 mm contiguous slice images of the chest without IV contrast, for which we adapted our CAD method. Nevertheless, the method we present is not data dependent and can be used for HRCT scans as well.

Considering 2-D computation of b-scale scenes, one may doubt if the algorithm can be extended into 3-D. Based on our observations on appearance and location of TIB patterns over the lung regions and experiences on feature extraction in 3-D, as we stated previously, TIB patterns rarely extend in depth direction for more than a few slices due to constraints of low-resolution imaging direction. Therefore, there is no significant classification rate changes in 3-D; however, there is an increase in computational cost. Nevertheless, 3-D b-scale encoding and feature extraction for a similar pattern detection problem or the same problem with high-resolution images (with thinner slice thickness compared to low-resolution CT images) can readily be combined and used with similar accuracies reported in this study.

Number of large and small b-scale patterns might *perhaps* be used to identify other type of abnormality patterns such as GGO and consolidations where we expect to have more large b-scale patterns than small b-scale patterns. Therefore, as an extension of this study, we will tune our proposed methodology with different types of abnormalities to generalize the CAD systems for infectious lung diseases in general.

Our proposed method is capable of detecting and quantifying TIB patterns very accurately as validated by the statistical tests compared to the expert annotations (i.e., ground truth). Therefore, both in detection and quantification steps, the proposed CAD system will highly possibly be helpful for clinicians as a second opinion tool in routine clinical examinations.

## VI. Concluding Remarks

In this study, we have proposed b-scale-based binary classification approach for automatic TIB pattern detection and quantification from chest CTs. The proposed system integrates 1) fast localization of candidate TIB patterns through b-scale filtering and scale selection, and 2) combined shape and textural features to identify TIB patterns. Note that texture-based recognition methods offer a complementary view to shape-based methods; therefore, the integration of spatial information and the proposed shape features achieves high detection rates. Moreover, our proposed local shape features illustrate the usefulness of the invariant properties, Willmore energy in particular, to analyze TIB patterns in chest CT. We have also compared computer scoring of the proposed CAD system with subjective visual grading. A high correlation between objective (CAD) and subjective (visual grading) scores is obtained, which implies highly satisfactory accuracy of the proposed CAD system.

## Supplementary Material

Color versions of one or more of the figures in this paper are available online at http://ieeexplore.ieee.org.
